# Depression, anxiety and health-related quality of life in paediatric intracranial germ cell tumor survivors

**DOI:** 10.1186/s12955-021-01911-9

**Published:** 2022-01-12

**Authors:** Wenyi Lv, Bo Li, Jin Feng, Li Chen, Xiaoguang Qiu, Shuai Liu

**Affiliations:** 1 Department of Radiation Oncology, Beijing Tiantan Hospital, Capital Medical University, Beijing, 100070 China; 2 Beijing Neurosurgical Institute, Capital Medical University, Beijing, 100070 China

**Keywords:** Intracranial germ cell tumours, Tumour survivors, HRQoL, Depression, Anxiety

## Abstract

**Background:**

Little is known about depression and anxiety among paediatric intracranial germ cell tumour (iGCT) survivors. We aimed to evaluate the risk factors associated with depression, anxiety and health-related quality of life (HRQoL) in paediatric iGCT survivors.

**Methods:**

We recruited 200 iGCT patients (and their parents) from Beijing Tiantan Hospital and assessed their HRQoL using the Paediatric Quality of Life Inventory (PedsQL) 4.0 Generic Core Scales. The Children’s Depression Inventory, Screen for Child Anxiety Related Emotional Disorders, and Symptom Checklist 90 were used to evaluate depression and anxiety. The results were analysed based on disease recurrence, tumour location and treatment strategies.

**Results:**

Survivors with recurrent tumours had worse HRQoL scores than those with non-recurrent tumours. Patients with tumours involving both the suprasellar and basal ganglia regions had the worst HRQoL scores. A large proportion of survivors had depression or anxiety. Both depression and anxiety scores were highly correlated with the HRQoL emotional functioning scores. The parent proxy-reports (PPR) and child self-reports were highly correlated in all domains.

**Conclusions:**

This study demonstrated the clinical factors affecting paediatric iGCT survivors’ depression, anxiety, and HRQoL. Therefore, psychological interventions should be implemented. It also suggests that the PedsQL PPR would be helpful for routine screening.

## Plain English summary

Intracranial germ cell tumours (iGCT) are relatively rare brain tumours that predominantly occur in paediatric patients. Since the survival rate of patients with iGCT has been improving and the burden of long-term morbidity is substantial, promoting the health-related quality of life (HRQoL) and reducing depression and anxiety among its survivors is of critical concern. Although many studies have been published on factors related to the HRQoL of iGCT survivors, little is known about their long-term emotional function. This study demonstrated the clinical factors that affect depression, anxiety, and the HRQoL and the associations between the HRQoL and depression/anxiety in 200 paediatric iGCT patients. We found that patients with recurrent tumours had a worse HRQoL. Tumour locations also played a critical role in that tumour involving the basal ganglia were associated with worse outcomes. A large proportion of paediatric iGCT survivors had depression or anxiety in all the groups. Depression/anxiety scores were highly correlated with the HRQoL emotional functioning scores. In addition, we found that the PedsQL parent proxy report is appropriate in most cases when it is not possible to obtain child self-reports. These findings provide evidence to support the optimization of treatment strategies for iGCTs, such as the provision of psychological interventions.

## Background

Intracranial germ cell tumours (iGCT) are relatively rare brain tumours. In Asians, iGCT account for 8–14% of all paediatric central nervous system tumours, with an incidence rate that is several times higher than that of Europe and the US (0.5–3%) [1, 2]. The 5-year survival rates of germinomas exceed 90% and those of non-germinomatous germ cell tumours (NGGCTs) are reported to range from 40 to 70% [3]. Since the survival rate for iGCT has been improving and the burden of long-term morbidity is substantial, improving the health-related quality of life (HRQoL) and reducing the rates of depression and anxiety among its survivors is of critical importance [4, 5].

Depression and anxiety are associated with cancer [6]. Studies have shown that survivors of childhood cancer are at higher risk of depression and anxiety compared to their healthy peers, and the prevalence of depression and anxiety symptoms increased many years after the completion of therapy [7]. Although some studies have been published on factors related to the HRQoL of iGCT survivors [8, 9], little is known about their long-term emotional function. The low incidence of iGCTs, the small number of follow-up cases and the lack of universal and easy-to-use screening scales have made such studies difficult to carry out [4].

Based on our clinical experiences and previous studies [10–12], we investigated if the clinical factors including tumor recurrence, locations, age and treatment strategies would affect patients' HRQoL, depression, and anxiety. Our aim was to comprehensively evaluate various aspects of the HRQoL, especially depression and anxiety, in paediatric iGCT survivors across different settings.

## Methods

### Participants

This study included 200 patients (along with their parents) who were diagnosed with iGCT between July 1994 and July 2017 at the Beijing Tiantan Hospital. The inclusion criteria were as follows: (1) diagnosis of iGCT without other brain diseases and the age at first diagnosis of iGCT was less than 18 years. (2) above 5 years of age at neuropsychological evaluation, (3) at least 2 years of survival after initial diagnosis and (4) completion of treatment at least a year prior to follow-up. The parents who spent the most time with the child were asked to complete the survey when both parents were available. Since some patients or parents declined to participate in the follow-up sessions or only completed part of the follow-up scales due to poor health status, busy work/school schedules, lack of interest, or death, the number of participants included in the different measurements varied (see Table 1). After obtaining informed consent from the participants, assessments were conducted in Chinese, and the tests were administered either in person or via mail. The current study was approved by the ethics committee of the Beijing Tiantan Hospital.

**Table 1:** Demographic and clinical characteristics

	n	%		n	%
Total participants	200	100	*Treatment method (non-recurrent)*		
Recurrence			Irradiation type		
Yes	10	10.0	Local	3	1.7
No	180	90.0	WBI	138	76.6
*Clinical data (non-recurrent)*			CSI	39	21.7
Gender			Radiation dose, Gy		
Female	56	31.1	Low (< 50)	114	63.3
Male	124	68.9	High (≥ 50)	66	36.7
Diagnosis			Surgery type		
GCT	131	72.8	No surgery	134	74.4
NGGCT	49	27.2	Biopsy only	9	5.0
β-HCG range			Definitive surgery	37	20.6
Normal			Chemotherapy		
High	105	58.3	Yes	177	98.3
AFP range	75	41.7	No	3	1.7
Normal			*Depression/anxiety*		
High	156	86.7	Child depression		
Tumor location	24	13.3	Healthy (CDI < 19)	86	92.5
Suprasellar region			Depression (CDI ≥ 19)	7	7.5
Pineal region	50	27.7	Child anxiety		
Basal ganglia	43	23.9	Healthy (SCARED < 23)	80	74.8
Bifocal origins A^a^	30	16.7	Anxiety (SCARED ≥ 23)	27	25.2
Bifocal origins B^a^	30	16.7	Adult depression		
Dissemination	5	2.8	Healthy (SCL-90 sub-score < 2)	35	68.6
	22	12.2	Depression (SCL-90 sub-score ≥ 2)	16	31.4
			Adult anxiety		
			Healthy (SCL-90 sub-score < 2)	41	80.4
			Anxiety (SCL-90 sub-score ≥ 2)	10	19.6

GCT, germ cell tumour; NGGCT, non-germinoma germ cell tumour; WBI, whole brain irradiation; CSI, craniospinal irradiation; CDI, children’s depression inventory; SCARED, screen for child anxiety related emotional disorders; SCL-90, symptom checklist 90 revised

^a^Bifocal origins A: pineal and suprasellar regions. Bifocal origins B: suprasellar region and basal ganglia regions

Regimens of radiotherapy for the non-recurrent iGCT patients comprised: (1) Whole-brain irradiation of 24.0–30.6 Gray (Gy) followed by a boost to the tumour region for the total dose of 36.8–60.0 Gy; (2) Craniospinal irradiation (CSI) of 23.0–36.1 Gy with a boost to the tumour region for the total does of 34.2–58.8 Gy; (3) A few patients only received radiotherapy to the primary tumour site (n = 3). Patients were recommended to complete 4–6 cycles of platinum-based chemotherapy.

### Assessment tools

The HRQoL was assessed using the Chinese version of the Paediatric Quality of Life Inventory (PedsQL) 4.0 Generic Core, which showed good psychometric properties [13]. Age-appropriate versions were provided to each participant. Both parent proxy report (PPR) and child self-report (CSR) scores were obtained for young children (age 5–7), children (age 8–12), teens (age 13–18), young adults (age 18–25), and adults (age 26 and above). PedsQL scores range from 0 to 100, with higher scores indicating better HRQoL. The total scale score (TSS), physical health summary score (PHSS), and psychosocial health summary score (PsHSS) were calculated. Emotional, social, and school/work functioning were measured and reported as sub-scores [14]. Previous studies identified the average TSS for PPR and CSR to be 82.70 and 83.84, respectively, among healthy children, as compared to 63.40 and 71.74 among children with brain tumours [15].

The presence and severity of depressive symptoms in children and adolescents (aged 9–17 years) were assessed using the Children’s Depression Inventory (CDI), which comprises a 27-item self-report questionnaire, each scored on a scale from 0 to 2. Total scores range from 0 to 54, with higher scores indicating more severe depression. CDI scores of 19 or higher were considered clinically meaningful to indicate significant depressive symptoms [16]. For children aged 9–17 years, anxiety was assessed using the Screen for Child Anxiety Related Emotional Disorders (SCARED) questionnaire, which includes 41 items, each comprising three statements. Total scores range from 0 to 82, with higher scores indicating more severe anxiety. A total score of 23 or higher indicates the presence of anxiety disorder [17].

In young adults aged 18 years and above, symptoms of depression and anxiety were assessed using the Symptom Checklist 90 Revised (SCL-90), a 90-item self-reported psychometric instrument that measures nine symptom dimensions: somatization, interpersonal sensitivity, obsessive–compulsive symptoms, depression, anxiety, hostility, phobic anxiety, paranoid ideation, and psychoticism. Dimension scores of 2 or higher were considered clinically meaningful to indicate psychological problems. Its reliability and validity have been previously established, and it is widely used to measure depression and anxiety in both research and clinical practice [18].

### Statistical analyses

Data were analysed using IBM SPSS Statistics version 25. The test results were represented based on the age-related norms published in the test manuals. Two-sample independent t-tests or Mann–Whitney U tests were used to compare participants’ depression, anxiety, and HRQoL according to tumour recurrence, treatment method, sex, serum clinical markers (β-HCG and AFP), and diagnosis. The overall effects of tumour location on all outcomes were explored using the one-way analysis of variance (ANOVA). Pearson’s correlation tests were conducted to detect the associations between the age at diagnosis, the number of years since treatment, the present age, irradiation dosage and outcome measures. Associations between the scales were assessed using Pearson’s correlation tests. Stepwise multiple linear regression analysis was applied to determine the significant predictive factors of HRQoL, allowing for entry and removal at the 0.05 and 0.10 level. For all analyses, the threshold for statistical significance was set at *p* < 0.05.

## Results

Table 1 lists the characteristics of the participants. The mean age at diagnosis was 11 years (range 3–17 years), and the mean age at neuropsychological evaluation was 16 years (range 5–39 years). The median follow-up time was 5 years (range 1–24 years). Of the 200 patients, 20 experienced tumour recurrence. Among the non-recurrent patients, the average total radiation dose was 46.3 Gy (range 34.2–60.0 Gy), and approximately 98.3% of the patients received chemotherapy, with a median number of six chemotherapy cycles. For the HRQoL of all patients, the participants’ average TSS values for PPR and CSR were 71.71 and 73.45, respectively.

Table 2 presents a comparison of the HRQoL, depression, and anxiety between patients with recurrent and non-recurrent iGCTs. Survivors with recurrent tumours had poorer HRQoL outcomes than non-recurrent tumours in the TSS, PHSS, PsHSS, emotional and school/work functioning domains of PedsQL PPR and TSS, PsHSS and emotional and school/work functioning domains of PedsQL CSR. Children survivors with recurrent iGCTs had significantly higher depression scores than those with non-recurrent tumours. No significant differences were observed between the groups in child anxiety scores and adult depression or anxiety scores. Among the non-recurrent patients, 4.7% and 23.5% of the children exhibited depression and anxiety, respectively, and 31.0% and 19.0% of adults exhibited depression and anxiety, respectively. Among the patients with relapse, 37.5% and 44.4% of the children exhibited depression and anxiety, respectively, and 33.3% and 22.2% of adults exhibited depression and anxiety, respectively.

**Table 2:** Overall outcomes by tumor recurrence

	Non-recurrence Mean (SD)	Recurrence Mean (SD)	*P*
PedsQL (PPR)			
Participants	152	18	
Total	72.95 (17.03)	61.29 (17.89)	**0.007**
Physical	76.73 (19.04)	64.24 (25.77)	**0.042**
Psychosocial summary emotional	70.93 (17.92)	59.72 (17.66)	**0.013**
Social	75.39 (16.77)	61.94 (19.64)	**0.006**
School/work	75.00 (22.72)	66.39 (21.54)	0.088
	62.40 (22.29)	50.83 (21.44)	**0.024**
PedsQL (CSR)			
Participants	124	15	
Total	74.72 (17.67)	62.97 (17.27)	**0.013**
Physical	77.17 (19.01)	66.46 (23.75)	0.095
Psychosocial summary emotional	73.41 (18.53)	61.11 (15.82)	**0.009**
Social	75.81 (18.25)	60.67 (18.11)	**0.006**
School/work	77.82 (22.34)	68.67 (19.22)	0.050
	66.61 (21.97)	54.00 (16.60)	**0.014**
Depression/anxiety			
Participants	85	8	
Child depression (CDI)	7.53 (5.34)	15.63 (11.16)	**0.034**
Participants	98	9	
Child anxiety (SCARED)	13.88 (10.77)	23.22 (15.71)	0.061
Participants	42	9	
Adult depression (SCL-90)	2.24 (2.85)	1.79 (0.82)	0.323
Adult anxiety (SCL-90)	1.69 (1.51)	1.51 (0.86)	0.725

Bold:* p* < 0.05

PedsQL, Pediatric Quality of Life Inventory 4.0 Generic Core Scales; PPR, parent proxy report; CSR, child self-report

Table 3 presents the HRQoL, depression, and anxiety grouped by tumour location in non-recurrent patients. Statistical analysis revealed significant differences in HRQoL based on the tumour location in the TSS, PHSS, social and school/work functioning domains of PedsQL PPR and TSS, PHSS, PsHSS, and social and school/work functioning domains of PedsQL CSR. The HRQoL was worst in patients with bifocal origins B (suprasellar region and basal ganglia) tumours and best in patients with pineal region tumours. Child anxiety scores and adult depression scores differed significantly according to tumour location. There were no significant differences in the child depression scores and adult anxiety scores.

**Table 3:** Overall outcomes by tumor location

	Suprasellar Mean (SD)	Pineal Mean (SD)	Basal Ganglia Mean (SD)	Bifocal A^a^ Mean (SD)	Bifocal B^a^ Mean (SD)	Dissemination Mean (SD)	*P*
PedsQL (PPR)							
Participants	40	37	27	25	5	18	
Total	71.55 (17.14)	77.00 (15.68)	68.84 (20.11)	77.22 (14.19)	50.87 (11.23)	74.09 (14.45)	**0.014**
Physical	74.30 (20.75)	83.28 (16.24)	71.30 (20.38)	82.13 (16.23)	54.38 (23.66)	75.52 (13.82)	**0.005**
Psychosocial summary emotional	70.08 (17.30)	73.65 (17.36)	67.53 (21.20)	74.60 (15.53)	49.00 (10.51)	73.33 (16.40)	0.053
Social	70.25 (17.36)	76.62 (16.83)	79.26 (16.45)	77.60 (15.15)	62.00 (10.37)	79.17 (16.74)	0.074
School/work	76.00 (21.55)	76.62 (20.85)	65.93 (28.89)	81.60 (20.75)	55.00 (17.68)	79.44 (17.31)	**0.044**
	64.00 (21.79)	67.70 (19.35)	57.41 (25.92)	64.60 (18.54)	30.00 (19.69)	61.39 (22.48)	**0.011**
PedsQL (CSR)							
Participants	37	28	22	21	2	14	
Total	77.41 (16.22)	79.54 (13.91)	69.17 (23.61)	72.10 (14.12)	36.96 (4.61)	76.01 (16.16)	**0.009**
Physical	77.70 (18.77)	84.15 (11.87)	70.45 (25.24)	77.23 (16.24)	32.81 (2.21)	78.57 (15.10)	**0.003**
Psychosocial summary emotional	77.25 (16.50)	77.08 (16.26)	68.48 (24.30)	69.37 (15.51)	39.17 (5.89)	74.64 (17.30)	**0.030**
Social	77.16 (15.66)	78.39 (19.25)	78.41 (19.30)	69.76 (19.78)	62.50 (17.68)	73.93 (18.83)	0.456
School/work	82.84 (17.93)	82.50 (17.02)	68.64 (30.79)	75.00 (21.68)	30.00 (7.07)	80.71 (18.28)	**0.004**
	71.76 (20.49)	70.36 (18.35)	58.41 (27.92)	63.33 (16.53)	25.00 (7.07)	69.29 (22.61)	**0.015**
Depression/anxiety							
Participants	28	22	14	15	2	4	
Child depression (CDI)	6.32 (4.60)	7.32 (5.62)	8.57 (6.32)	8.20 (4.74)	18.00 (1.41)	5.75 (3.40)	0.060
Participants	31	27	17	17	1	5	
Child anxiety (SCARED)	14.42 (11.35)	18.63 (11.72)	8.00 (7.31)	12.24 (9.55)	7.00	11.80 (6.54)	**0.039**
Participants	6	8	7	10	1	10	
Adult depression (SCL-90)	1.56 (0.47)	1.47 (0.51)	1.11 (0.95)	4.97 (4.96)	2.31	1.32 (0.72)	**0.022**
Adult anxiety (SCL-90)	1.35 (0.27)	1.35 (0.25)	1.16 (0.84)	2.97 (2.68)	2.40	1.20 (0.39)	0.062

Bold:* p* < 0.05

^a^Bifocal origins A: pineal and suprasellar regions. Bifocal origins B: suprasellar region and basal ganglia regions

In non-recurrent patients, the older the age at diagnosis, the worse the emotional performance, including CSR emotional scores (r = − 0.227, *p* = 0.011), SCARED (r = 0.207, *p* = 0.041) and CDI (r = 0.364, *p* < 0.001); the older the age at evaluation, the worse the emotional and social performance, including CSR emotional functioning scores (r = − 0.240, *p* = 0.007), CSR social function scores (r = − 0.177, *p* = 0.049) and CDI (r = 0.274, *p* = 0.011). With the increase in the number of follow-up years, the social scores of non-recurrent patients worsened (CSR social scores, r = − 0.182, *p* = 0.043).

No statistically significant differences in sex, serum clinical markers (β-HCG and AFP), diagnosis, surgery type, radiation area and radiation dose were observed in HRQoL, depression, or anxiety. Tumour markers were grouped and analysed according to normality or abnormalities. All the factors (tumour recurrence, tumour location, age at diagnosis, age at evaluation, follow-up time, sex, serum clinical markers, diagnosis, surgery type, radiation area and radiation dose) were included in the stepwise multiple linear regression model. The analysis showed that tumour recurrence was significant predictive factor for TSS of both PedsQL PPR (Unstandardized Coefficients B = − 15.438, SE = 5.309, t = − 2.908, *p* = 0.004, R^2^ = 0.050) and CSR (Unstandardized Coefficients B = − 14.176, Std. Error = 5.835, t = − 2.429, *p* = 0.016, R^2^ = 0.043).

Additionally, we investigated the associations between different treatment strategies and HRQoL in patients with tumours in the same region. Patients with suprasellar tumours showed statistically significant differences. Specifically, the TSS of the PedsQL PPR was significantly different (*p* = 0.003) through non-surgical treatment (M = 73.13, SD = 14.57), surgical resection (M = 33.70, SD = 27.67) and biopsy (M = 80.98, SD = 11.53); and the CSR’s TSS of the NGGCTs group (M = 66.85, SD = 18.53) were significantly lower than those of the germinoma group (M = 80.32, SD = 14.55; *p* = 0.029). Notably, the NGGCT group received a significantly higher radiation dose than the germinoma group (M = 50.3 Gy vs M = 41.8 Gy, *p* < 0.001).

There were strong correlations between depression/anxiety and HRQoL emotional functioning scores. PedsQL CSR emotional functioning scores were strongly related to child depression scores (CDI, r = − 0.489, *p* < 0.001), child anxiety scores (SCARED, r = − 0.468, *p* < 0.001), adult depression scores (SCL-90 depression sub-scores, r = − 0.433, *p* = 0.007) and adult anxiety scores (SCL-90 anxiety sub-scores, r = − 0.454, *p* = 0.005). PedsQL PPR and CSR scores were also strongly correlated with Pearson correlation coefficients, from 0.723 (emotional scores), 0.757 (social scores), 0.794 (school/work scores), 0.807 (PsHSS), 0.814 (PHSS) and 0.831 (TSS).

## Discussion

This study examined a large cohort of 200 paediatric iGCT survivors’ depression, anxiety, and HRQoL. It also investigated the clinical factors that affected the HRQoL, and first demonstrated the role of tumour relapse, special tumour locations (bifocal lesions, dissemination), depression and anxiety. These findings bring more consideration to the treatment strategies of iGCT and the importance of considering paediatric iGCT survivors' HRQoL.

Twenty (10.0%) of the 200 participants with iGCT experienced relapse. Many studies have investigated the HRQoL in patients with brain tumours. Unfortunately, very few studies have investigated the impact of recurrent brain tumours, especially in paediatric patients. Our findings indicated that survivors with relapse showed worse HRQoL scores than non-relapse survivors. Moreover, there were higher proportions of depression and anxiety in survivors with tumour recurrence than in those without relapse. This result was similar to that of a previous study by Giovagnoli, who found that patients with recurrent brain tumours manifested high degrees of state and trait anxiety as well as depression, along with poor affective well-being, role and leisure and sharing [10]. The authors believe that the extensive damage of HRQoL after brain tumour recurrence may be caused by many factors, such as poor physical condition, extreme life changes, worries about one’s disease and the future, and the side effects of repeated treatment.

Tumour location plays a critical role in the HRQoL of the iGCT survivors. Among non-recurrent patients, patients with tumours in both the suprasellar and basal ganglia had the worst HRQoL, followed by those with basal ganglia tumours. Compared with tumours in the pineal or suprasellar regions, basal ganglia iGCTs often cause more severe motor dysfunction and neurocognitive impairment, such as cognitive, learning, memory, language, and behavioural impairment [19–21], leading to lower HRQoL [22]. Suprasellar tumours impair children’s hormone levels and can lead to dysfunctions of the hypothalamus and pituitary gland, which could explain the trends in cognitive function decline observed in a previous study [11]. Hypothalamic-pituitary axis dysfunction is the most common complication of childhood central nervous system tumours and cranial radiotherapy. Systematic follow-up and early endocrine consultation are critical to counteract this effect [23].

We further analysed the treatment of iGCTs located in the same region. Among patients with suprasellar tumours, pathological type (NGGCTs) and definitive surgery negatively affected their HRQoL and mental health. Similar results were not obtained in other sites, which may be due to the fact that the suprasellar region is more vulnerable than other regions and is more susceptible to surgery and high-dose radiotherapy [11]. Moreover, tumours in other regions may cause much greater brain damage than that caused by treatment, especially iGCT arising from the basal ganglia, which are often accompanied by ipsilateral atrophy of the basal ganglia, brainstem, and cortex. The ipsilateral hemiatrophy may be due to Wallerian degeneration caused by tumour cells infiltrating the fibre tracts [24]. Brain damage caused by tumours may obscure the influence of different treatment strategies. In addition, we included patients with a large time period of diagnoses (> 20 years) and shifts in treatment, such as the decreasing trend of radiation dose and radiation range. This may also have an impact on the results.

Interestingly, older patients at diagnosis seem to have more emotional problems. The age at diagnosis was not significantly associated with physical health scores but was significantly correlated with emotional scores, and these problems did not seem to be resolved with the treatment of the disease. In non-recurrent paediatric survivors, the older the age at diagnosis, the worse the emotional performance; the older the age at evaluation, the worse the emotional and social performance. With the increase in the number of follow-up years, the social scores worsened. After the diagnosis of a paediatric brain tumour, patients may be more prone to acute adaptive function damage and often demonstrate internalisation, anxiety, depression, withdrawal, and consequent social problems [25, 26]. Psychological intervention should be implemented to counteract patients’ perceptions of their disease and their condition as hopeless [27].

Similar to most previous studies, no statistically significant differences in HRQoL were observed according to sex or serum clinical markers (β-HCG and AFP) [28, 29].

HRQoL emotional functioning scores were highly correlated with depression and anxiety scores. Although depression and anxiety are complex, they are important contributing factors to poor HRQoL [12]. Besides treating diseases, understanding children’s mental status, monitoring depression and anxiety, and providing psychological support are also critical to improving their quality of survival [30].

Consistent with Kuhlthau’s study, the PPR and CSR scores were highly correlated in all domains, indicating that PPR scores are appropriate in most cases when it is not possible to obtain CSR scores [8]. Consistent with other studies, in most measurements, parents reported worse HRQoL scores than their self-reported children [31]. This may be because parents have more concerns about their children's physical condition, disease recurrence and long-term side effects.

The present study has some limitations. First, clinical data were collected retrospectively and at a single point in time. Since clinical data on the patients’ depression, anxiety and HRQoL before treatment were unavailable, the results could not distinguish changes caused by the tumour itself, the subsequent treatment or interactions between the two, neither did it reveal progressive changes in the HRQoL. Additionally, due to incomplete clinical data, the study did not discuss some factors that were closely related to the prognosis of patients with iGCT, such as hormone deficiency and visual impairment. These clinical factors and other factors regarding parent informants (e.g. education and socio-economic status) should be carefully considered in future studies. Second, although to the best of our knowledge, this is the largest data series involving paediatric iGCT patients, the sample size of this study remains limited. iGCT develops in various locations, and the location of tumours has a significant impact on patients’ HRQoL and mental status. When exploring the prognostic factors of iGCT located in the same region, this study’s sample size was particularly small. Thus, the findings may not be generalizable to all iGCT survivors. Third, some iGCT survivors declined to participate in the study due to poor mental/physical health, lack of interest, or death, and those who consented to participate may have been more health-conscious. Finally, the feasibility of using the HRQoL emotional score as a screening scale for patients with depression or anxiety needs to be further confirmed by a study with a larger sample.

## Conclusions

This retrospective study generated valuable data that expands our understanding of the influence of recurrence, tumour location, and treatment method on paediatric iGCT survivors’ depression, anxiety, and HRQoL. Further, PedsQL PPR scores are appropriate in most cases when it is not possible to obtain CSR scores. Early depression and anxiety screening and psychological intervention would be helpful for improving the outcomes of iGCT survivors and should be a standard of care in patients' follow-up.

## Data Availability

The data that support the findings of this study are available from the corresponding author upon reasonable request.
